# The Educational Retrograde at St. Louis

**Published:** 1904-09

**Authors:** 


### Editorial.

### THE EDUCATIONAL RETROGRADE AT ST. LOUIS.

The indignation expressed at the action of the—so-called—Association of Faculties at St. Louis has been widespread since the publication in our August number. This is not confined to the residents of this country, but is being voiced by those interested in dental education in other lands, and this will be intensified as time gives opportunity for expression.

The blow fell, and dental education in America suffered a collapse that will require years to overcome and re-establish confidence.

The faction that passed the absurd resolutions at St. Louis were well assured that its action could not be sustained, but there was sufficient intelligence to be aware that, however iconoclastic this might be, it would have the result of creating a panic in the ranks of those favorable to a continuance of four years. The active workers in this faction had, apparently, no higher incentive, and, now that they have accomplished their purpose, they must feel great gratification, and can leisurely proceed to destroy each other without let or hinderance from any organization; for have they not "repealed all rules or parts of rules" interfering with them? The motive that actuated this action on their part may be right from their point of view, but it means, to many of them, college destruction. Those who so enthusiastically adopted these resolutions, in the expectation that they could be a law unto themselves, will assuredly find that the protection of the National Association of Dental Faculties meant something, and was not a mere resolution to be rescinded at pleasure, but a shield that enabled each member to feel that the rules adopted for the protection of each college would be rigidly enforced. This confidence, destroyed at St. Louis, cannot be restored, and while the Association of Faculties may retain a nominal existence, its power for good has ceased, not, however, through a decision of a majority of its members, but through the determination of the minority to rule or ruin. Is it possible to place confidence in the future of such a membership?

The present is full of doubt, and leading dental educators are viewing the prospect with anxiety and with grave uncertainty. The dental colleges of this country will doubtless all accept this retrograde action, and matriculate students this year for three years and postpone further changes until State boards and Legislatures have decided to act.

At this writing it is impossible to determine the influences that may be brought to bear in this direction in the near future. The probabilities are that no positive action will be taken this year, and it is a question whether anything can be suggested that will improve the present chaotic condition.

It is humiliating to feel that dental education in the United States will have nothing to show at the two World's Congresses,—the International Dental Federation and the International Dental Congress. The delegates to these have been invited to see the fruits of a dentistry that has steadily advanced through a century, and they will see some of this, but they will also see a profession incapable of maintaining a standard worthy of their respect, or that of those who sent them, representing all nationalities of the world.

If these iconoclasts at St. Louis supposed that their action would be permanent, they must be convinced by this time that they were positively mistaken. The element in this country solicitous for a more perfect standard will not be thus lightly repulsed. It is an element not governed by mercenary considerations, and, in consequence, possesses a vitality that will long outlast the dollar motive, that, at best, is intrinsically weak and short lived. While the blow dealt at dental education from the house of its supposed friends has been a severe one, it will not be lasting, for the best in professional life cannot remain submerged, but will rise above the temporary deluge of selfishness into the clearer atmosphere of a truer educational work and life.

